# The Appetite Suppressant D-norpseudoephedrine (Cathine) Acts *via* D1/D2-Like Dopamine Receptors in the Nucleus Accumbens Shell

**DOI:** 10.3389/fnins.2020.572328

**Published:** 2020-10-16

**Authors:** B. Kalyanasundar, Claudia I. Perez, Benjamin Arroyo, Mario Gil Moreno, Ranier Gutierrez

**Affiliations:** Laboratory of Neurobiology of Appetite, Department of Pharmacology, Center for Research and Advanced Studies (CINVESTAV), Mexico City, Mexico

**Keywords:** anorexigenic drugs, food intake, locomotor activity, weight loss, nucleus accumbens, obesity

## Abstract

D-norpseudoephedrine (NPE), also known as cathine, is found naturally in the shrub *Catha edulis* “Khat.” NPE has been widely used as an appetite suppressant for the treatment of obesity. Although it is known that NPE acts on α1-adrenergic receptors, there is little information about the role of dopamine receptors on NPE’s induced anorectic and weight loss effects. Equally untouched is the question of how NPE modulates neuronal activity in the nucleus accumbens shell (NAcSh), a brain reward center, and a pharmacological target for many appetite suppressants. To do this, in rats, we characterized the pharmacological effects induced by NPE on weight loss, food intake, and locomotion. We also determined the involvement of dopamine D1- and D2-like receptors using systemic and intra-NAcSh antagonists, and finally, we recorded single-unit activity in the NAcSh in freely moving rats. We found that NPE decreased 24-h food intake, induced weight loss, and as side effects increased locomotor activity and wakefulness. Also, intraperitoneal and intra-NAcSh administration of D1 and D2 dopamine antagonists partially reversed NPE’s induced weight loss and food intake suppression. Furthermore, the D1 antagonist, SCH-23390, eliminated NPE-induced locomotion, whereas the D2 antagonist, raclopride, only delayed its onset. We also found that NPE evoked a net activation imbalance in NAcSh that propelled the population activity trajectories into a dynamic pharmacological brain state, which correlated with the onset of NPE-induced wakefulness. Together, our data demonstrate that NPE modulates NAcSh spiking activity and that both dopamine D1 and D2 receptors are necessary for NPE’s induced food intake suppression and weight loss.

## Introduction

Obesity is currently a pandemic affecting more than 650 million people worldwide. Although obesity is primarily treated with exercise and diet, appetite suppressants can aid in weight loss (Wing and Hill, 2001; Joo and Lee, 2014; Brett, 2019). Amphetamine was the first appetite suppressant widely used in humans, but in the late 1960s, it was restricted because of its highly addictive properties (Harris et al., 1947; Stowe and Miller, 1957; Sharp et al., 1962; Stark and Totty, 1967; Drevets et al., 2001). Subsequent appetite suppressants were mainly amphetamine congeners but with less intense properties (Zelger and Carlini, 1980; Kalix and Khan, 1984; Balint et al., 2009; Khan et al., 2012). These drugs exert their pharmacological effects by stimulating the release of norepinephrine, serotonin, and dopamine (DA) via uptake inhibition (Baumann et al., 2000; Drevets et al., 2001; Rothman et al., 2001; Broening et al., 2005). The most commonly prescribed appetite suppressants since 1959, are phentermine and diethylpropion (Bray, 2000; Kushner, 2018). However, there is very little information about their mechanism of action, especially on their central effects. To fill this gap, recently, we found that mild stimulants, including diethylpropion, phentermine, and bupropion, suppressed food intake, induced weight loss, and modulated neural activity in the nucleus accumbens shell (NAcSh) (Kalyanasundar et al., 2015; Perez et al., 2019), a brain region with robust dopaminergic innervation involved in feeding, sleep, and locomotor behavior (Kelley et al., 2005; Palmiter, 2007; Tellez et al., 2012). In contrast to the idea that they mainly act via norepinephrine and serotonin neurotransmitters, we found that D1- and D2-like DA receptor antagonists greatly attenuated their anorectic and weight loss effects (Kalyanasundar et al., 2015). However, there is a scarcity of information about D-norpseudoephedrine (NPE), an appetite suppressant introduced in the 1970s, used for weight reduction. Thus, here we have extended our studies to NPE.

The leaves of “Khat” contain several active compounds. Still, the most potent is cathinone [S-(−)-cathinone], followed by two diastereoisomers: cathine [1S,2S-(+)-norpseudoephedrine, abbreviated as NPE] and norephedrine [1R,2S-(−)-norephedrine] (Kalix and Khan, 1984; Balint et al., 2009). Cathinone and its less potent metabolite NPE are referred to as natural amphetamines (Kalix, 1992). However, and despite that, they are structurally related to amphetamine; they exhibit fewer addictive properties (specially NPE), according to the World Health Organization (Eddy et al., 1965; Zelger and Carlini, 1980; Eisenberg et al., 1987; Kalix et al., 1990; Toennes et al., 2003). Eisenberg et al. (1987) showed that rats treated with NPE exhibited increased locomotor activity and reduced food intake at doses of 10–50 mg/kg. Moreover, NPE has diminished potency relative to cathinone but shown to have a longer duration of action (Peterson et al., 1980; Zelger and Carlini, 1980; Pehek et al., 1990). Nevertheless, over the past decades, NPE has received very little experimental attention; only a few clinical studies have examined NPE as an appetite suppressant for the short-term treatment of obesity (Greenway, 1992; Richert, 2011; Lemieux et al., 2015; Onakpoya et al., 2016). Recently, the long-term (24 weeks) efficacy and safety for NPE were studied in humans, with promising results (Hauner et al., 2017). Nonetheless, and despite its widespread use, their evoked behavioral and neuronal effects remain poorly understood.

This study aimed to increase our knowledge about the mechanism of action of NPE and its effects evoked in the brain. We found that blockage of DA receptors partially reversed NPE-induced pharmacological effects. Our results confirm and further extend the idea that most, if not all, appetite suppressants of the phenethylamine class exert their anorectic effects via NAcSh’s D1- and D2-like receptors (Kalyanasundar et al., 2015).

## Materials and Methods

### Animals

A total of 62 male Sprague–Dawley rats ∼250–350 g were used for all experiments. Future studies should evaluate the effects of NPE in female rats. Animals were housed individually and had *ad libitum* access to food and water except during multichannel recordings or when locomotion was measured in the open field (see below). Room temperature was maintained at 21 ± 1°C, with 12/12 h light–dark cycle (0600–1,800 h). All procedures were approved by the CINVESTAV institutional animal care and use committee.

### Drugs and Chemicals

The NPE hydrochloride was kindly provided by Productos Medix (Mexico). R(+)–SCH-23390 hydrochloride (SCH) and S(−)-raclopride (+)-tartrate salt (RAC) were obtained from Sigma–Aldrich (Mexico). These compounds were dissolved in physiological saline (Sal) (0.9% NaCl) and administered intraperitoneally (i.p.) in a volume of 1 ml/kg or 2.5 μg/0.5 μL per hemisphere in the intra-NAcSh infusion (see below).

### Dose–Response Effects of NPE on Weight Loss and Food Intake

To determine whether NPE influences the rats body weight and food intake, these variables were measured approximately at the same time once daily 20 min before the experiment’s start. Rats were injected with Sal (*n* = 3), 10 mg/kg NPE, 20 mg/kg NPE, 40 mg/kg NPE, or 80 mg/kg NPE, namely, NPE10, NPE20, NPE40, and NPE80, respectively (*n* = 4/group). Animals were individually housed and received 100 g allotment of standard rat chow (Purina Mexico) per day. Behavioral experiments were carried out between 1,600 and 1,800 h (i.e., 2 h prior to the commencement of the rat’s active phase). The daily changes in body weight and food intake were expressed in grams relative to the baseline (BL, i.e., the values recorded 20 min before the experiment’s start, in the first injection day).

### Locomotor Activity in the Open Field

All locomotor effects were measured using Ethovision XT10 (Noldus Information Technology, the Netherlands) (Perez et al., 2019). Our setup recorded up to six open field arenas simultaneously (40 L × 40 W × 30 H cm). The arenas were placed together in two rows (3 × 2) and a CCD camera (IDS camera, Germany) with a uEye Cockpit software recorded with a top view and 15-fps resolution. After 3 days of habituation to the open field, animals were injected with their corresponding treatment once daily and then were placed in an open field for 90 or 120 min. The videos were analyzed using the center body mass, tracking the position of the animal as “x” and “y” coordinates to compute the total distance traveled (cm). Two videos were lost and thus not included into the analysis. They corresponded to days 2 and 4 for the same animals; one rat for NPE10, NPE20, and NPE40 groups.

### Effects of Systemic Administration of DA D1- and D2-Like Receptor Antagonists on NPE-Induced Pharmacological Effects

To determine the contribution of D1- and D2-like receptors on NPE-induced behavioral effects, we injected SCH or RAC antagonists i.p. over 7 consecutive days. The antagonist concentrations were selected based on previous studies showing that doses 1.5 mg/kg (SCH) and 0.5 mg/kg (RAC) antagonized the locomotor effects induced by amphetamine, methamphetamine, and diethylpropion (Broening et al., 2005; Wright et al., 2013; Kalyanasundar et al., 2015). Based on our dose–response experiment, the NPE’s effective dose (ED_50_) that leads to weight loss was 20 mg/kg. Thus, we used this dose for all subsequent experiments. Animals were divided into six groups: Sal + Sal (*n* = 4), SCH + Sal (*n* = 3), RAC + Sal (*n* = 3), Sal + NPE (*n* = 4), SCH + NPE (*n* = 4), and RAC + NPE (*n* = 4). The first name indicates the initial i.p. injection of the DA antagonist, and the following name represents the second i.p. injection received. Body weight and food intake (both in grams) were measured daily 20 min before each experiment’s start. Rats were habituated to the open field for 2 days (data not shown). During the treatment, the rats were daily introduced in the open field during 0–45 min to record the basal locomotor activity (with no injection), and then at 45 min, they received the first i.p. injection with either Sal or two antagonists (either SCH or RAC). Then at 60 min, they received the second i.p. injection (either Sal or NPE), and their locomotor activity was monitored for 1 additional hour.

### Intra-NAcSh Infusions of DA Antagonists

#### Cannula Implantation

To evaluate the role of the DA receptors located in the NAcSh on the NPE-induced changes in weight loss, feeding, and locomotion, we performed intra-NAcSh infusions of DA antagonists. Animals were anesthetized using a cocktail of ketamine (100 mg/kg) and xylazine (8 mg/kg). For the bilateral intra-NAcSh infusions, two holes were drilled at the following coordinates: AP +1.4 mm; L ± 1 mm from bregma. Stainless-steel guide cannulas (0.63 diameter × 11 mm in length) aimed at the NAcSh were bilaterally inserted 5.5 mm DV relative to bregma. Two screws served as anchors in the skull bone, and the whole assembly was cemented with dental acrylic. A stylus was inserted into the cannula to prevent clogging, and it was removed before each daily microinjection. For all animals, enrofloxacin (10 mg/kg, i.p.) and Baytril (5%, i.p.) were administered for 3 days after surgery, and they were allowed to recover for 7 days.

The microinjections were performed via a 30-gauge stainless-steel injector, protruding 1.0 mm from the guide cannula’s tip, connected via a Teflon tube to a 10-μL glass microsyringe (Hamilton 80366) attached to a microinfusion pump (KD scientific- KDS200 series). A total volume of 0.5 μL (0.33 μL/min) per hemisphere of RAC or SCH was infused once daily for 7 days. The injector was left into the cannula for 1 additional minute to allow drug diffusion (Gutiérrez et al., 2003).

Following the same procedures used in systemic experiments, 18 naive animals were assigned to six groups, each of n = 3: Sal + Sal, SCH + Sal, RAC + Sal, Sal + NPE, SCH + NPE, and RAC + NPE. Body weight and chow food intake were measured daily just before placement in the open field. Then, rats were introduced in the open field during 0–45 min (with no injection). At 45 min, animals were briefly removed and microinjected with either Sal, SCH, or RAC (2.5 μg/0.5 μL) into the NAcSh, and then they were returned to the open field. Next, an i.p. injection of either Sal or NPE (20 mg/kg) was given at 60 min. The rat’s locomotor activity was recorded for 1 additional hour.

### Electrophysiology

#### Surgery

Animals were anesthetized using a cocktail of ketamine (100 mg/kg) and xylazine (8 mg/kg), and implanted with an i.p. catheter following the protocol described by Perez et al. (2019). After the catheter’s surgery, we inserted an electrode array in the NAcSh following previously described methods (Tellez et al., 2012). Briefly, a movable 4 × 4 microwire array (tungsten wires of 35 μm in diameter) was unilaterally implanted in the right NAcSh at the following coordinates: AP = 1.4, L = ± 1, and DV = 7.5 mm from bregma. One stainless-steel screw was soldered to a silver wire (203 μm) and implanted on the cerebellum’s surface served as ground. Finally, the electrode array was anchored to the skull using dental acrylic and two more screws. To maintain the patency, catheters were flushed daily with Sal (0.9%). Seven days after surgery, rats were habituated for 2 days to the operant box. A polyethylene tube, 30 cm in length, was connected to the catheter attached to a syringe outside the box and was manually operated to infuse the drug (either Sal or NPE) non-invasively during the neuronal recordings.

#### Single-Unit Recordings in the NAcSh of Freely Moving Rats

Recordings (19 sessions from three rats) were performed using a multichannel acquisition processor (Plexon, Dallas, TX). During recordings, rats were placed in an operant box enclosed in a sound-attenuating cubicle equipped with a webcam. Each session lasted for 3 h and consisted of three epochs: BL (0–1 h), Sal (1–2 h), and treatment (NPE: 2–3 h; 20 mg/kg). Voltage signals were sampled at 40 kHz and digitalized at 12-bit resolution. The action potentials were band-passed filter (154 Hz to 8.8 kHz). Only single neurons with action potentials with a signal-to-noise ratio larger than 3:1 were analyzed. Action potentials were identified online using a voltage–time threshold window and the three principal components contour template algorithm of neuron’s wave shape (Gutierrez et al., 2010). Action potentials were then sorted using offline sorter software (Plexon Dallas, TX). No food or water was available during recordings to avoid unintended modulations induced by chewing or licking and those induced by satiety. Thus, the intention was primarily to record the pharmacological effects induced by NPE. The NAcSh’s local field potentials (LFPs) were amplified 1,000×, filtered 0.7–300 Hz, and digitized at 1 kHz using a digital acquisition card (National Instruments, Austin, TX). They were used to compute the brain state hypnograms (see below).

#### Statistical Analyses for Behavior

All data are presented as mean ± SEM. Statistical differences both within- and between-subjects’ factors were assessed by two-way repeated-measures analysis of variance (RM ANOVA), followed by Tukey *post hoc* analysis using GraphPad Prism 7. Complete statistical analyses on body weight, food intake, and locomotor activity can be found in Supplementary Table 1.

#### Calculation of NPE’s ED_50_ for Weight Loss

We calculated the effective dose 50 (ED_50_) on body weight loss using the equation *f* = min + ((max–min)/[1 + (ED_50_/*X*)^Hill slope]), where *f* is the expected response to a given dose (X), min and max are the lowest and highest weight loss, and the ED_50_ is the dose at which 50% of the subjects are expected to show the desired response using the program developed for Gadagkar and Call (2015).

#### Neurons That Increase or Decrease Firing Rates After Administration of NPE

All analyses were performed using MATLAB toolboxes and homemade custom scripts. After the injection of NPE, a significant change in firing rate was identified using a Kruskal–Wallis test, considering a significance level α < 0.05. Neurons exhibiting an increased firing rate after treatment relative to BL epoch were classified as “increased,” whereas neurons with reduced activity were named “decreased.” We used a χ^2^-test to assess differences in the percentage of neurons modulated.

### Principal Component Analysis for Population Activity Trajectories

#### Preprocessing

All neuronal responses were concatenated in a single n × m matrix *D*, where rows were ***n*** = neurons, and ***m*** = columns were firing rates binned at 1-s size. The neuronal’s responses were smoothed with a Gaussian kernel (σ = 100 ms) as follows:

$$f\,\left(x|t\,s,\sigma \right)=\frac{1}{\sigma \,\sqrt{2\,\pi }}*e^{\frac{-{\left(x-t\,s\right)}^{2}}{2\,\sigma^{2}}}$$

where ***ts*** were timestamps for every spike per neuron, ***x*** was a vector containing values to construct each Gaussian kernel (e.g., ts - 3σ: 10 ms: ts + 3σ), and ***σ*** was kernel standard deviation. Then, all neural responses were downsampled in bins of 30-s size. The neural responses were *z* scored as follows:

$$f\,R_{\text{z-score}}=\frac{f\,R-\mu }{\sigma }$$

where *fR* was a vector containing the firing rate of a given neuron, μ is the mean firing rate, and σ is the standard deviation of the firing rate of a given neuron. The standard deviation and mean firing rate were calculated from the BL (time 0–1 h).

#### Principal Component Analysis

To understand the neural dynamics of the population NAcSh’s responses, we used principal component analysis (PCA) to calculate the linear combinations of the population activity, capturing the most variance in the averaged population responses. The population activity was projected onto two axes, corresponding to the first two principal components to describe both temporal dynamics and the relationships across the different task’s epochs (BL, Sal, and NPE) present in the population activity trajectories. This analysis was computed in MATLAB using the following line code:

[∼, scr, ∼] = pca(D′, ‘Rows’, ‘complete’);

plot(scr(:,2), scr(:,1), ‘-o’, ‘linewidth’, 2);

axis tight; grid on; xlabel(‘PC 2’); ylabel(‘PC 1’);

where ***D′*** was the transposed matrix of *z* scored neural responses, and ***scr*** were the principal components, which correspond to the first two principal components explaining the most variance.

#### Hypnograms: The LFP’s Brain State Map

For hypnograms, behavioral states were assigned using information obtained from the LFP as outlined in Gervasoni et al. (2004) and Tellez et al. (2012). In brief, after the elimination of segments with amplitude saturation, a sliding window Fourier transform was applied to each LFP signal to calculate two spectral amplitude ratios (0.5–20/0.5–55 Hz and 0.5–4.5/0.5–9 Hz for ratios 1 and 2, respectively). PCA was then applied to these ratios obtained from all LFP channels, and the PCAs were used as the overall ratios measure. These measures obtained for each second of data were further smoothed with a Hanning window (20 s length). Finally, the two PCAs of the spectral ratios were plotted against each other to construct a two-dimensional (2-D) state space where the density of points reflects the relative abundance of the different brain states. Rapid eye movement (REM) sleep was not included in this analysis because animals spent very little time in REM state (data not shown). The final 2-D brain state maps were selected and validated after visual inspection of animals’ behavior in the video within three behavioral states: slow-wave sleep (SWS), quiet wake (qW), and active wake (aW) (Tellez et al., 2012).

#### Histology

At the end of the experiments, rats were injected with pentobarbital sodium (150 mg/kg i.p.) and perfused with PBS, followed by 4% paraformaldehyde. Brains were placed in a 10% sucrose (vol/vol) solution for 24 h, with sequential increases in sucrose concentration until reaching 30% in 72 h. The brain slices (50 μm) were stained with cresyl violet to verify the cannula locations and recording sites (Supplementary Figure 1).

## Results

### Behavioral Effects

#### NPE-Induced Weight Loss

**FIGURE 1:**
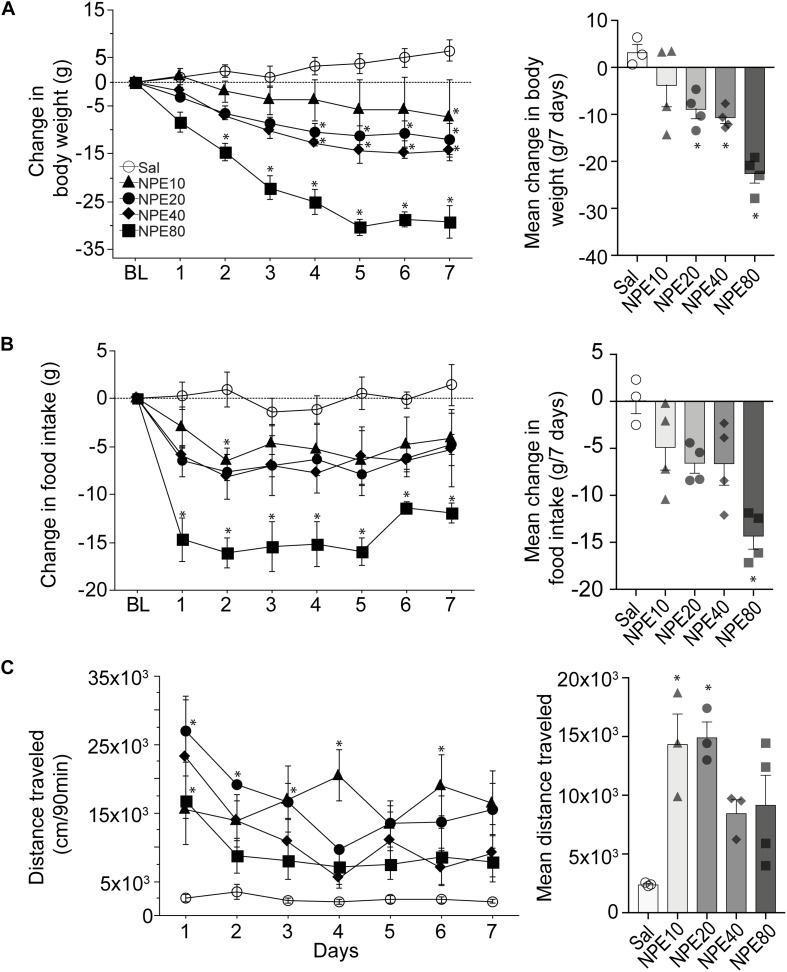
D-Norpseudoephedrine (NPE) leads to weight loss, suppresses food intake, and NPE increases locomotor activity. **(A)** *Left panel:* Change in body weight (g) relative to baseline day (BL) over 7 consecutive injections of saline (Sal) or NPE at 10-, 20-, 40-, and 80-mg/kg doses (NPE10, NPE20, NPE40, and NPE80, respectively). The horizontal dotted line represents no change in body weight relative to BL. *Right panel*: Average body weight loss over 7 consecutive days of treatment. **(B)** *Left panel*: Change in chow intake (g) over 7 days. *Right panel*: Average change in chow intake. **(C)** *Left panel*: Locomotor activity induced by different doses of NPE. *Right panel*: Mean distance traveled follows inverted-U shape response. Bars represent the mean ± SEM. *p < 0.05 significantly different from saline-treated rats.

To determine the efficacy of NPE, we measured the body weight of rats over 7 consecutive days. Figure 1A, left panel, shows the change in body weight relative to initial BL weight after administering either control Sal or one of the following doses of NPE; 10, 20, 40, and 80 mg/kg. We found that Sal-treated rats gained body weight over the 7-day treatment period. Statistical analysis demonstrated a significant main effect of doses on body weight [RM ANOVA; *F*_(__4, 14)_ = 13.1, *p* = 0.0001; time (days): *F*_(6, 84)_ = 21.32, *p* < 0.0001, and doses × time interaction: *F*_(24,84)_ = 4.3, *p* < 0.0001]. In contrast, NPE20, NPE40, and NPE80 treatments resulted in a significantly greater weight loss than the control Sal group across days (all *p*_*s*_ < 0.05). We also plotted the average change in body weight in Figure 1A, *right panel*. A *post hoc* analysis unveiled that NPE20 and NPE40 induced a similar weight loss (*p* > 0.9), whereas NPE20, NPE40, and NPE80 induced more weight loss than the Sal group (^∗^*p* < 0.05).

#### Administration of NPE Suppressed Food Intake

To study the NPE-induced anorectic effects, we measured the chow food intake. Figure 1B, left panel, shows the daily change in food intake (in g) relative to BL (Figure 1B, left panel). As expected, the administration of Sal did not change food intake, because values remained around zero. In contrast, NPE10, NPE20, and NPE40 inhibited food intake with a similar magnitude (−4.9 ± 0.8, −6.6 ± 0.4, and −6.6 ± 0.8 g, respectively), whereas the highest NPE dose 80 mg/kg achieved the maximum reduction −14.3 ± 0.7 g (Figure 1B, right panel [RM ANOVA; main effect of doses: *F*_(4, 14)_ = 7.9, *p* = 0.0015; time (days): *F*_(6, 84)_ = 3.3, *p* = 0.005, and no significant doses × time interaction: *F*_(24, 84)_ = 0.7, *p* = 0.8]. A Tukey *post hoc* analysis unveiled that NPE at doses of 80 mg/kg was significantly different to Sal-treated rats (*p* < 0.05); whereas the lowest doses exhibited a non-significant trend suppressing food intake.

#### NPE Increased Locomotor Activity

Previous studies reported that acute administration of appetite suppressants in rats also stimulates locomotor activity (Reimer et al., 1995; Rothman and Baumann, 2006; Kalyanasundar et al., 2015; Perez et al., 2019). We then asked whether NPE modulates this behavior (Figure 1C, left panel). As expected, Sal administration (control) did not change locomotor activity. In contrast, we observed that locomotor activity was significantly higher after injection of NPE compared to the control group [RM ANOVA; main effect of doses: *F*_(4, 11)_ = 6.4, *p* = 0.006; time (days): *F*_(6, 66)_ = 4.3, *p* = 0.0009, and doses × time interaction: *F*_(24, 66)_ = 1.9, *p* = 0.01]. Note that rats treated with NPE10 and NPE20 exhibited greater locomotor activity than the Sal-treated rats (*p* < 0.05), whereas the highest doses NPE40 and NPE80 showed a reduced activity compared to the lowest doses (Figure 1C, right panel). NPE’s increased locomotor activity tends to follow a dose-dependent curve of inverted-U shape, suggesting that normal locomotion was compromised in large doses, as shown in Thiel and Dressler (1994). In rats, our dose–response curve for the weight loss over 7 days of treatment with NPE yielded an effective dose (ED_50_) of 20 mg/kg. Therefore, we used the ED_50_ dose for all subsequent experiments.

#### NPE-Induced Weight Loss, Food Intake Suppression, and Locomotion Were Attenuated by Systemic Injection of DA D1 and D2 Receptor Antagonists

**FIGURE 2:**
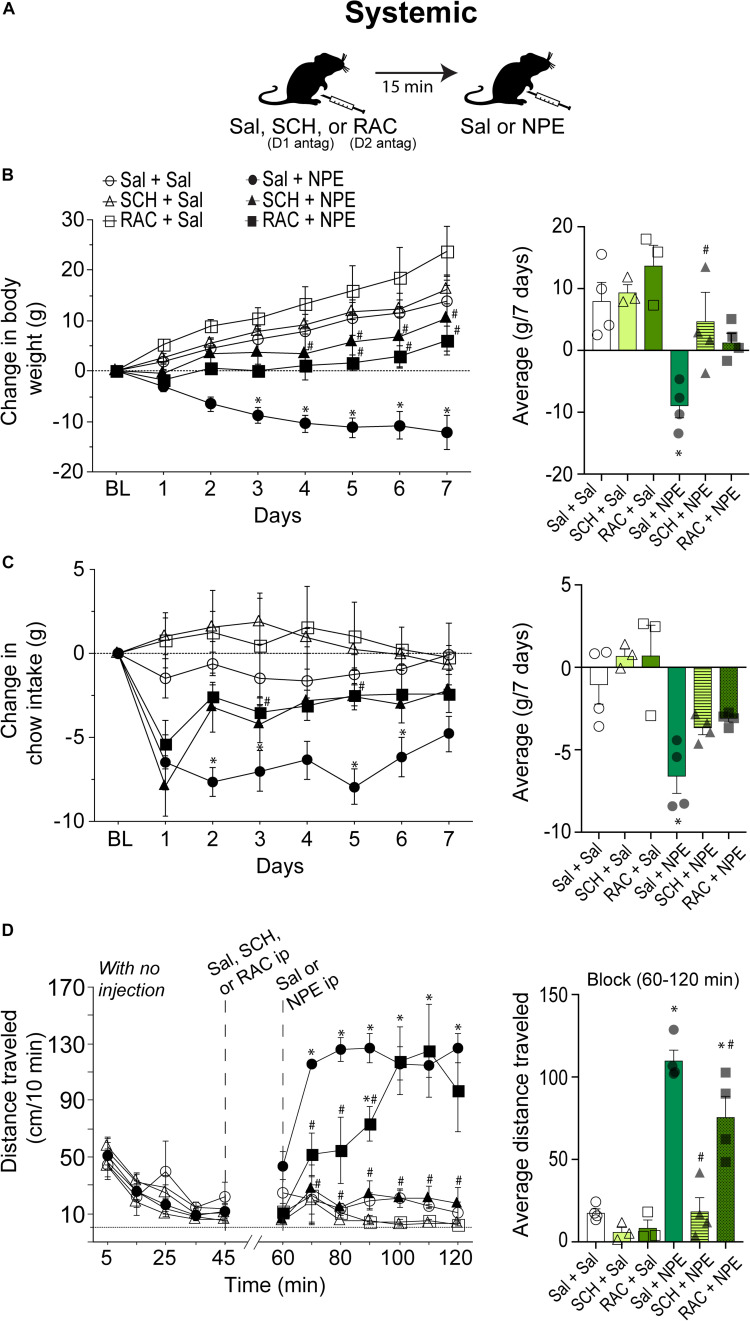
Intraperitoneal injection of either D1 (SCH23390- SCH) or D2-like receptor [raclopride (RAC)] antagonists partially reversed NPE -induced behavioral effects. **(A)** Scheme of i.p. injections. **(B)** *Left panel:* Change in body weight over 7 days relative to BL (first day of injection). NPE was given in a fixed dose of 20 mg/kg. *Right panel*: The average change in body weight. **(C)** *Left panel* shows the change in chow intake (g). *Right panel*: The average change in food intake. **(D)** *Left panel*: Distance traveled (cm/10 min) for a period with no injection (interval 0–45 min), and the evoked distance after NPE or Sal (>60 min). The first i.p. injection (Sal, SCH, or RAC) was given at 45 min, which is 15 min before the second injection at 60 min (Sal or NPE, note: the break-in *x*-axis at 45–60). *Right panel:* Average distance traveled. ^∗^*p* < 0.05 significantly different than in Sal + Sal. ^#^*p* < 0.05 compared to Sal + NPE group.

We then went to determine whether the NPE’s induced pharmacological effects depend on DA D1 and D2 receptors. Thus, either D1 (SCH; SCH23390 1.5 mg/kg) or D2 receptor antagonist RAC (0.5 mg/kg) were administered systemically 15 min before the i.p. injection of NPE or Sal. Figure 2A depicts the drug administration protocol. We found a significant treatment effect [*F*_(5, 16)_ = 7.2, *p* = 0.001], days [*F*_(6, 96)_ = 24, *p* < 0.0001], and a significant interaction between factors [*F*_(30, 96)_ = 4.9, *p* < 0.0001]. Figure 2B, left panel, shows the change in body weight over 7 consecutive days. The control group continued to gain body weight over time (Sal + Sal: 8 ± 1.3 g, Figure 1B, right panel). Likewise, the DA D1 antagonists alone (i.e., SCH + Sal) did not affect the weight gain compared to the control group (Sal + Sal: 8 ± 1.3 g vs. SCH + Sal: 9.4 ± 1 g; *p* = 0.99, n.s.). Although RAC + Sal exhibited a slighter increase in body weight gain (13.7 ± 1.7 g) than rats treated with Sal, the difference did not achieve statistical significance (*p* = 0.76, n.s.). In contrast, Sal + NPE leads to a robust and significant weight loss (−9.0 ± 0.9 g, *p* = 0.0063). Most importantly, blockade of DA D1 receptors attenuated NPE-induced weight loss, because SCH + NPE (4.7 ± 1.7 g; black triangles) was significantly different than Sal + NPE group (*p* = 0.03). Though the RAC + NPE group (1.5 ± 0.7 g; black squares) did not reach statistical significance (*p* = 0.143 n.s. for the full effect) (Figure 2B, right panel), the day-by-day analysis uncovered that RAC + NPE group attenuated NPE-induced weight loss until days 5–7 (see # in Figure 2B, left panel, black squares). Our data demonstrate that D1-like (and in less degree D2-like) receptors are critical players for NPE-induced weight loss.

We measured the 24 h food intake. Figure 2C, left panel, shows the change in food intake over 7 days of treatment. RM ANOVA found a treatment main effect: *F*_(5, 16)_ = 9.0, *p* = 0.003; no time effect (days): *F*_(6, 96)_ = 1.5, *p* = 0.1, and no significant treatment × time interaction: *F*_(30, 96)_ = 1.2 *p* = 0.1. The Sal + Sal, SCH + Sal, and RAC + Sal were not significantly different among the three control groups. In contrast, Sal + NPE group exhibited a significant reduction in chow intake compared to Sal + Sal (see ^∗^ right panel). Note that on the first day DA antagonists did not eliminate the initial food intake suppression induced by Sal + NPE. However, after the second and subsequent days, blockade of DA receptors partially reversed Sal + NPE-induced food intake suppression. Accordingly, SCH + NPE and RAC + NPE were not statistically different from Sal + Sal group (*p* = 0.35 and *p* = 0.6). These results demonstrated that systemic administration of DA antagonists partially attenuated NPE-induced anorectic effects.

We also measured NPE’s induced locomotion effects. Figure 2D, left panel, shows the daily locomotor activity. During the 0–45 min period (with no injection), all groups exhibited a similar distance traveled (<70 cm/10 min: all *p*_*s*_= n.s.). Likewise, Sal + Sal group showed no increase in locomotor activity between BL and after Sal injection (0–45 min: 28.5 ± 5.5 cm/10 min; 60–120 min: 17.7 ± 2.4 cm/10 min). In contrast, from 60 to 120 min, the comparison among the six groups showed a significant effect of treatment on locomotion [*F*_(5, 16)_ = 32.7, *p* < 0.0001] and time [*F*_(6, 96)_ = 5.2, *p* = 0.0001] and significant interaction between factors [*F*_(30, 96)_ = 3.3, *p* < 0.0001]. After systemic administration of DA antagonists alone, rats showed no increase in locomotion; in fact, they showed a rather decreased in locomotor activity compared to Sal + Sal group (SCH + Sal: 6.1 ± 2.4 cm/10 min; RAC + Sal: 8.5 ± 3 cm/10 min, and Sal + Sal: 17 ± 2.4 cm/10 min, all *p* = n.s.). In contrast, Sal + NPE–treated rats exhibited increased locomotor activity in comparison to Sal + Sal. The D1 receptor antagonist SCH completely reversed the locomotion induced by NPE (SCH + NPE vs. Sal + NPE; *p* < 0.0001; Figure 2D *right panel*). The D2 antagonist RAC mainly delayed the NPE-induced locomotion (*p* = 0.04; RAC + NPE). Thus, these data suggest that the NPE-induced locomotion depends more on DA D1 than D2 receptors, suggesting an important role of D1 receptors on NPE-induced locomotor effects.

#### Blockade of D1- and D2-Like Receptors Directly in the NAcSh Attenuated the NPE-Induced Behavioral Effects

**FIGURE 3:**
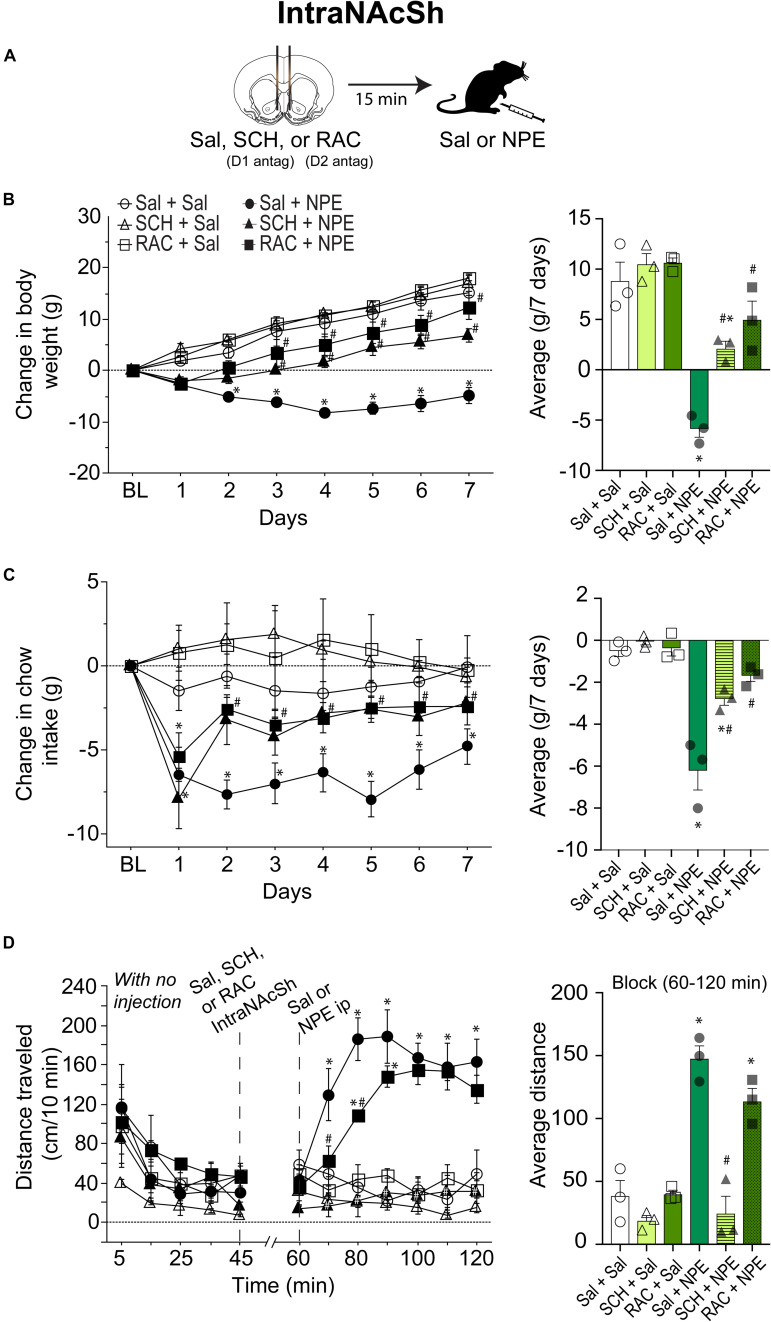
Role of intra-NAcSh dopamine D1- or D2-like receptors on NPE’s induced behavioral effects. **(A)** The protocol of drug administration: intra-NAcSh injection either Sal, or RAC, or SCH (at 45 min), followed by i.p. injection of either Sal or NPE (at 60 min, see panel **D**). **(B)** *Left panel:* Change in body weight during 7 consecutive days of treatment. *Right panel* averaged over 7 days. **(C)** *Left panel:* Change in chow intake (g) relative to BL. *Right panel*: The average change in chow intake. **(D)** *Left panel:* The distance traveled (cm/10 min) on a period with no injection (interval 0-45 min) and after injections (60–120 min). *Right panel*: Average distance. ^∗^*p* < 0.05 significantly different than in Sal + Sal. ^#^*p* < 0.05 compared to Sal + NPE group.

To evaluate the involvement of DA receptors located in the NAcSh for the NPE-mediated behavioral effects, we microinjected either D1 or D2 antagonists directly into the NAcSh, while rats received an i.p. injection of Sal or NPE (Figure 3A). Figure 3B, left panel, shows the change in body weight over 7 days. Statistical analysis found a significant effect of treatment [RM ANOVA; *F*_(5, 12)_ = 26.4, *p* < 0.0001; time (days): *F*_(6, 72)_ = 122.7, *p* < 0.0001; and interaction: *F*_(30, 72)_ = 8.5 *p* < 0.0001]. DA antagonists alone in the NAcSh did not affect body weight gain compared to the control group (Figure 3B, right panel: Sal + Sal: 8 ± 1.g; SCH + Sal: 9.4 ± 1 g; RAC + Sal: 13.7 ± 1.7 g). In contrast, the weight loss induced by NPE was significantly different than Sal + Sal (Sal + NPE; -5.8 ± 0.5 g; *p* < 0.0001). The weight loss induced by NPE (Sal + NPE) was significantly attenuated by intra-NAcSh infusion of either SCH (SCH+ NPE: weight gain; 2.1 ± 0.8 g; *p* = 0.0063) or RAC (RAC + NPE: 4.9 ± 1.2 g; *p* = 0.0005). Our data demonstrate that D1- and D2-like receptors in the NAcSh are involved in the NPE-induced weight loss.

We then evaluate their effects on food intake (Figure 3C). Direct infusion of DA antagonists into the NAcSh significantly attenuated the NPE-induced food intake suppression (Figure 3C, left panel) {effect of treatment [*F*_(5, 12)_ = 27.1, *p* < 0.0001], and days [*F*_(6, 72)_ = 22.1, *p* < 0.0001], and significant interaction [*F*_(30, 72)_ = 3.9, *p* < 0.0001]}. Briefly, food intake in the three control groups (Sal + Sal, SCH + Sal, and RAC + Sal) showed that they consumed the same amount of chow (i.e., values remained at the same level than BL, *p* = n.s.; Figure 3C, right panel). In contrast, the administration of NPE inhibited food intake compared to Sal + Sal (Sal + NPE; *p* < 0.0001), and its anorectic effects were attenuated by the infusion of DA antagonists. That is, rats treated with D1 or D2 antagonists (SCH + NPE or RAC + NPE groups) consumed more chow food than rats treated with Sal + NPE (all *p*_*s*_ < 0.01). These data show that NPE’s anorectic effects were markedly reduced by D1 and D2 receptor antagonism in the NAcSh.

The analysis of the locomotor activity further uncovered the major involvement of D1 DA receptors. Figure 3D, left panel, shows the distance traveled for each group. During 0 to 45 min, there were no significant differences between the control groups, except that the SCH + Sal group showed lower locomotor activity during the period with no injection (open triangle perhaps as a result of a carryover effect). In the interval 60 to 120 min, RM-ANOVA demonstrated a main effect of treatment [*F*_(5, 12)_ = 30.3 *p* < 0.0001], time [*F*_(6, 72)_ = 8.7, *p* < 0.0001] and interaction [*F*_(30, 72)_ = 6.5 *p* < 0.0001]. During this period, the antagonists administered alone, SCH + Sal (18.8 ± 2.6 cm/10 min), and RAC + Sal (39.9 ± 3.8 cm/10 min) failed to induce locomotor activity compared to Sal + Sal (38.5 ± 5.2 cm/10 min, all *p*_*s*_ = n.s. Figure 3D, right panel). In contrast, Sal + NPE group exhibited a robust locomotion (147.7 ± 12.4 cm/10 min) compared to Sal + Sal (*p* < 0.0001). We found that SCH + NPE (24.5 ± 4.9 cm/10 min) completely attenuated the locomotion induced by Sal + NPE (Figure 3D, left panel, *p* < 0.0001), whereas the RAC + NPE group only delayed the onset but did not reverse the locomotion induced by Sal + NPE (113.8 ± 10.6 cm/10 min; Figure 3D, right panel; *p* = 0.20). Again, these results demonstrated that DA D1-like receptors in the NAcSh are the major contributors for NPE-induced locomotion.

### Electrophysiology

### Characterization of NPE Induced Modulation of Neuronal Activity in the NAcSh

**FIGURE 4:**
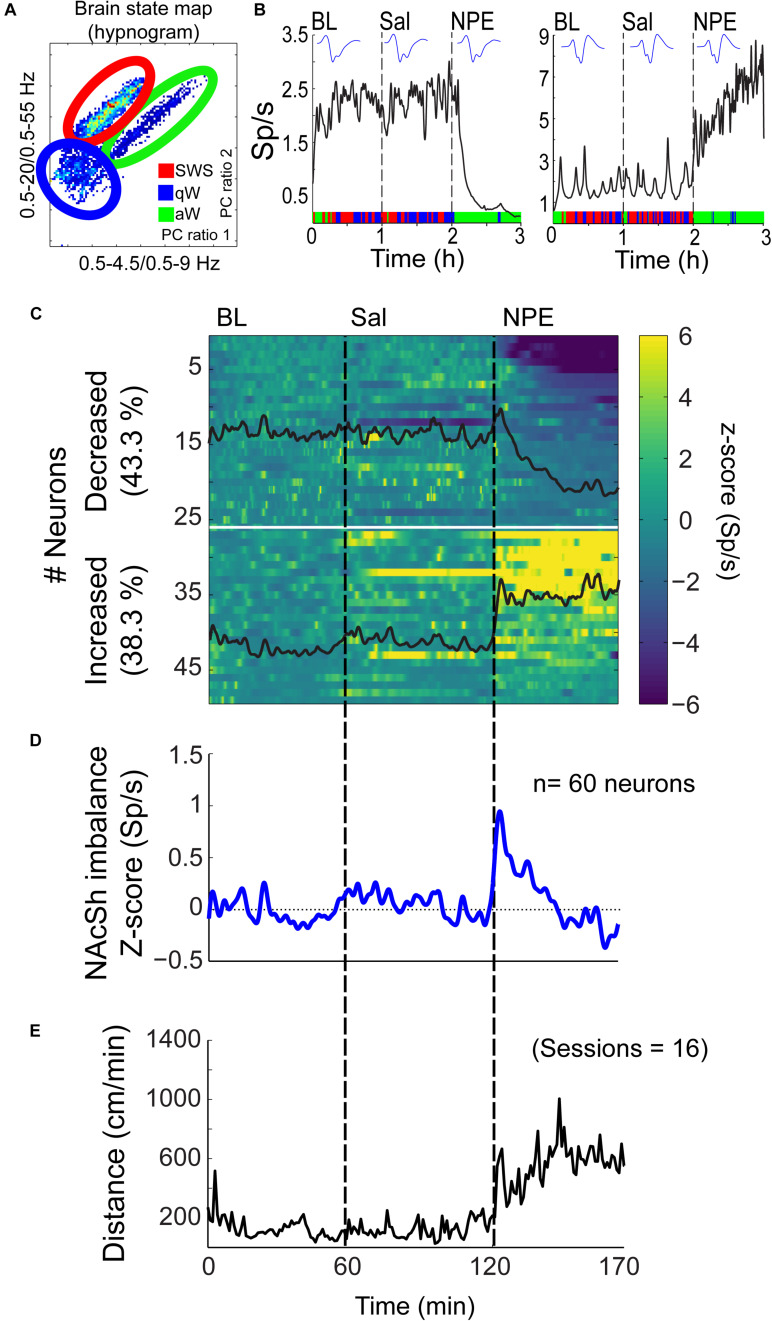
NPE modulates NAcSh spiking activity. **(A)** A brain state map (hypnogram) was computed from the local field potentials (LFPs) from the NAcSh, illustrating the three major behavioral states: slow-wave sleep (SWS), quiet awake (qW), and active awake (aW). Each dot represents the principal component (PC) ratio between two power intervals for each second of NAcSh’s LFP activity. Each dot falling into the red ellipsoid corresponded to periods when the animal was in SWS, and in blue and green, ellipsoids represent qW and aW brain states, respectively. **(B)** *Left panel*: An example of one neuron exhibiting decreased firing rates (spikes/s, Sp/s) after NPE injection. The action potential waveform did not change across the session. The color bar on the *x*-axis represents the SWS, aW, and qW states. *Right panel:* A typical neuron with increasing firing rates after NPE injection. **(C)** A color-coded peristimulus time histogram (PSTH) of NAcSh neuronal responses of 49/60 neurons recorded during BL, Sal, and NPE epochs (vertical black lines). The horizontal white line split neurons with decreased from increased neural responses. **(D)** The average population PSTH activity of all 60 NAcSh neurons recorded. **(E)** Average locomotor activity (cm/min) obtained across 16 recording sessions.

Given that the NAcSh receives strong dopaminergic input from dopaminergic neurons in the ventral tegmental area (Powell and Leman, 1976; Cauda et al., 2011), we demonstrated that the pharmacological effects of NPE were attenuated by both systemic and intra-NAcSh infusion of DA antagonists. Furthermore, to understand how NPE modulates NAcSh neural activity, we recorded single-unit activity after the injection of NPE. Figure 4A displays a brain state map obtained from NAcSh LFP’s oscillations. Each dot represents 1 s of LFP signal corresponding to a brain state mapping to a particular rat’s behavior. It clearly shows that animals exhibited three different behavioral states: (1) SWS, dots falling inside the red ellipsoid; (2) qW (blue); and (3) aW (green). Table 1 shows the time spent in each behavioral state as a function of epochs: BL period (BL, 0–1 h), Sal (1–2 h), and 20 mg/kg of NPE (2–3 h). Interestingly, after the injection of NPE, the predominant brain state changed from SWS to aW; animals spent most of the time on this active brain state (Figure 4B, see bottom for hypnograms of two different experiments). This is also evident in Table 1 in NPE’s epoch; animals rarely exhibited SWS (<1 min), because they stayed most of the time in the aW state reflecting insomnia [54.9 min; *F*_(2, 54)_ = 113.7, *p* < 0.0001]. At the single neuronal level, NPE evoked a tonic, and long-lasting, modulation in spiking activity. Figure 4B shows two representative modulatory responses induced by NPE and their corresponding brain state map (SWS, aW, and qW). The neuronal activity is depicted as a function of the following epochs: BL, Sal, and NPE, which are illustrated along with their waveform across the three epochs (see blue insets). Figure 4B, left panel, shows a representative neuron that reduced their firing rates after NPE administration relative to both BL and Sal period (BL: 2.17 ± 0.005 Sp/s; Sal: 2.28 ± 0.004 Sp/s; NPE: 0.44 ± 0.008, Sp/s). In contrast, the example shown in Figure 4B, right panel, comes from a neuron that increased activity after NPE. Specifically, the firing rate during the BL and Sal period was lower and similar (BL: 1.64 ± 0.008 Sp/s; Sal: 1.79 ± 0.007 Sp/s), but after NPE, it gradually increased (2–3 h: 5.78 ± 0.02 Sp/s).

**TABLE 1:** Time spent in each behavioral state across the epoch.

**Behavioral states**	**Time (min)**		
	**BL**	**Sal**	**NPE**
SWS	37.9 ± 0.9	34.9 ± 1.5	0.7 ± 0.2*
qW	7.9 ± 0.6	9.9 ± 0.8	3.1 ± 1.5
aW	13.4 ± 1.0	14.3 ± 1.8	54.9 ± 1.7*

Values are mean ± SEM, n = 19, *p < 0.05 significantly different from baseline (BL) and saline (Sal) epochs. SWS, slow wave sleep; qW, quiet wake; aW, active awake.

**FIGURE 5:**
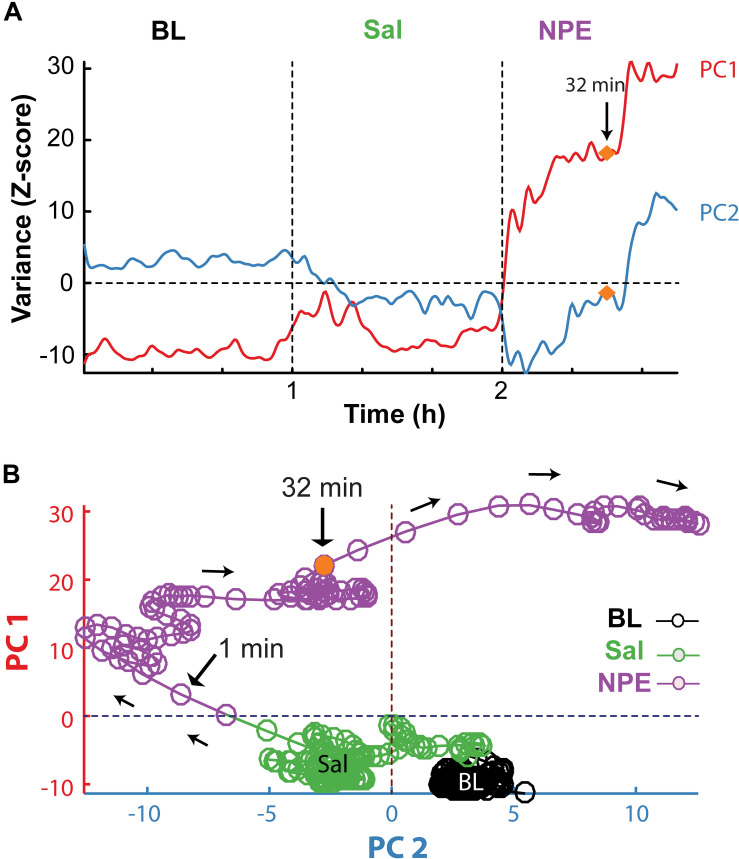
PCA population trajectory analysis unveil that NPE induced a dynamic pharmacological brain state. **(A)** The two first principal components as a function of time computed from the normalized NAcSh population PSTH activity. The first PC1 contains the contribution of neurons exhibiting increasing responses (PC1), whereas the second PC2 reflects the activity of decreasing responses after the onset of NPE. **(B)** Population trajectories projected into two-dimensional spaces, along the PC1 (increasing responses) vs. PC2 (decreasing responses). Each circle represents the population activity (in a 30 s bin size), and the color represents different epochs; BL (black circles), Sal (green circles), and NPE (purple circles). Note that neuronal activity in BL epoch remained around an attractor state. Likewise, activity slightly changed in the Sal epoch reflecting a non-stationary activity of the NAcSh. However, they again stayed in a new attractor state. In contrast, 1 min after administration of NPE, the population trajectories entered a different position in the PCA space. In both panels, the orange circle indicates 32 min after the onset of the NPE administration, where population trajectories changed once more.

Subsequently, we explored how NPE modulates NAcSh’s population activity. A total of 60 neurons were recorded from three rats in the NAcSh, while Sal and NPE were injected. Figure 4C displays the normalized NAcSh neuronal activity (only neurons significantly modulated by NPE are shown, *n* = 49/60) using a population color-coded PSTH (peristimulus time histogram), where yellow colors indicate responses above BL activity, and dark blue colors represent decreased responses. After NPE, 43.3% (26/60) of neurons were classified as decreased, while 38.3% (23/60) were increased [χ^2^_(1)_ = 0.64, *p* = 0.42, n.s.], and the other 18.3% (11/60) were non-modulated (Table 2 shows the average firing rate as a function of epochs). Overall NPE modulated 81.6% of NAcSh neurons. The average population activity of all recorded neurons exhibited an initial bias toward activation (Figure 4D). That is, the NAcSh population activity rapidly exhibited an activation imbalance reaching a maximum peak in about 1–2 min after NPE that returned to BL levels around 32 min (*z* scores crossed 0 again), despite that these rats exhibited the highest locomotion level at this time (Figure 4E) and some individual neurons still exhibited a gradual ramp-up activity for at least 1 h or more (e.g., Figure 4B, right panel). Thus, to gain a better understanding of the population dynamics induced by NPE, we performed a PCA to describe their population trajectories (Figures 5A,B). We found that the first PC1 explains the most variance and captures the increasing neuronal responses, whereas the second PC2 reflects the decreasing responses. By plotting the scores of the PC1 against PC2, in 30 s bin size increments, we can now observe that, during BL, most black circles fell within the same PCA subspace for nearly 1 h (see BL overlapping black circles in the scatter plot). During the Sal epoch activity, trajectories slightly moved, however, this change seem not to be related to Sal since it occurred minutes before Sal injection and because it remained stable in the new PCA subspace for nearly 1 additional hour (see Sal labeled overlaid in green circles). More interestingly, 1 min after NPE injection, NAcSh’s population activity entered in a non-physiological pharmacological brain state. Further at 32 min (orange circle and big arrow), it jumped again into a different and dynamic PCA trajectory, at the same time the average population activity had appeared to be returned to BL levels (see Figure 4D, i.e., excitation and inhibition seem to be canceling each other). Nevertheless, NPE-induced modulation at the single-unit level was sustained for at least an hour (Figure 4C), while the animal was awake and moving (Figure 4E). Thus, from the PCA analysis, it is evident that NPE induces a pharmacological brain state that correlated with wakefulness (Figure 5).

**TABLE 2:** Firing rate in each epoch.

**Type of response**	**Firing rate (Sp/s) per 60-min epoch**		
	**BL**	**Sal**	**NPE**
Decreased (26/60)	2.6 ± 0.004	2.4 ± 0.004	1.1 ± 0.009*
Increased (23/60)	2.4 ± 0.004	2.8 ± 0.004	4.2 ± 0.004*

Values are mean ± SEM. *p < 0.0001 comparisons to BL and Sal epoch.

## Discussion

Obesity has reached epidemic proportions worldwide. The current recommendations for the treatment of obesity and overweight include physical activity and reduced caloric intake. When behavioral intervention is not sufficient, pharmacotherapy is recommended (Derosa and Maffioli, 2012; Kushner, 2018; Brett, 2019). In the present study, we found that rats treated with NPE decreased food intake showed greater weight loss and more locomotion than Sal-treated rats. Most importantly, antagonism of both DA D1- and D2-like receptors, either systemic or intra-NAcSh, partially reversed NPE-induced behavioral effects. Electrophysiological recordings further uncovered that NPE evoked a strong modulation on NAcSh’s single-unit and population activity that correlated with the onset of the active awake brain state, indicative of insomnia. Our data, in rats, give further support of NPE as a robust appetite suppressant.

### NPE as a Robust Appetite Suppressant

NPE was originally used in the 1970s for the short-term treatment of obesity (Zelger and Carlini, 1980; Greenway, 1992; Richert, 2011). However, there is very little information about their behavioral and neuronal responses elicited in the NAcSh. We found that NPE has a significant weight-reducing effect for the doses tested, where intermediate doses (20 and 40 mg/kg) induced the same weight loss, but at 80 mg/kg NPE was more effective than the other doses (Figure 1). The reason for this phenomenon is not clear, but the same results were found in humans (Hauner et al., 2017). Moreover, we observed that NPE induced less tolerance over 7 days on food intake (Nencini et al., 1996) than other appetite suppressants such as diethylpropion and phentermine (Figure 1B; see also Kalyanasundar et al., 2015). Our results confirm previous studies showing that NPE decreased the food intake and could lead to weight loss in both rodents and humans (Zelger and Carlini, 1980; Eisenberg et al., 1987; Kalix, 1992; Hauner et al., 2017). Furthermore, our results also agree with the findings of Schechter (1990a), who found that rats trained to discriminate against the interoceptive cues produced by cathinone or amphetamine “generalized” to NPE. Likewise, acute tolerance, i.e., tolerance after a single dose, occurs when NPE is tested 24 h after cathinone or amphetamine administration (Schechter, 1990b). The “generalization” effect depends on DA release because CGS10746B, an inhibitor of presynaptic DA release, blocked this effect. Altogether, these results raised the possibility of dopaminergic signaling nature of the NPE’s cue and/or its production of tolerance (Pehek et al., 1990; Schechter, 1990a). Our findings confirm that DA D1/D2 receptors mediate NPE induced food suppression, which is in line with the idea that DA plays a major role in regulating food intake and caloric energy balance (Fernandes et al., 2020). Moreover, a state of DA dysregulation has been observed in obese rats (Geiger et al., 2009; Alsiö et al., 2010). Thus, it is tempting to propose these appetite suppressants may help to restore the lower dopaminergic tone observed in obese rats (Axel et al., 2010; Hansen et al., 2013). Taking together, the pharmacological and behavioral effects induced by NPE reflect the importance of DA signaling on feeding behavior.

### D1- and D2-Like DA Receptors Are Responsible for NPE’s Induced Locomotor Activity

Locomotion is a motor behavior often observed spontaneously in rodents and known to be induced by drugs such as DA agonists (Beninger, 1983; Liu et al., 1998; Schwienbacher et al., 2002). Here we demonstrated that NPE increased locomotor activity at 10 and 20 mg/kg doses, but at the high doses (40 and 80 mg/kg) reduced locomotor activity (Figure 1C). This inverted-U shape is a hallmark effect of amphetamine congeners on locomotion (Kalix, 1996; Perez et al., 2019). Our results are consistent with other studies demonstrating that drugs that enhance DA transmission increase locomotor activity or produce stereotypy depending on the dose (Daberkow et al., 2013). Likewise, amphetamine congeners increase the release of DA and stimulate locomotor activity at lower doses. However, at higher doses, locomotion is suppressed and replaced by stereotypy behavior in the form of head weavings (Segal and Mandell, 1974; Kalyanasundar et al., 2015; Perez et al., 2019). Behavioral studies, using systemic injections of drugs (amphetamine and cocaine), suggest that both DA D1- and D2-like receptors play an important role in locomotor activity (Dreher and Jackson, 1989; Baldo et al., 2002; Lecca et al., 2004; Knab et al., 2012; Moratalla et al., 2017). Moreover, the role of the NAc in motivated movement is well known (Meyer et al., 1993; O’Neill and Shaw, 1999; Baldo et al., 2002). For example, in rats, the administration of dopaminergic agonists promotes multiple behaviors, including locomotion, grooming, rearing, and stereotypy. Likewise, the infusion of DA or its agonists into the NAc enhances locomotor activity (Hoffman and Beninger, 1985; Dreher and Jackson, 1989; Meyer et al., 1993; O’Neill and Shaw, 1999; Baldo et al., 2002). Here we found that NPE increases locomotor activity, and the blockage of D1- and D2-like receptors, either systemic or into NAcSh, had a significant effect in NPE-induced locomotor activity (Figures 2D, 3D). Similar results were found by O’Neill and Shaw (1999) demonstrating that a systemic administration of D1 antagonist SCH 23390 reduced the locomotion induced by amphetamine, cocaine, and SKF82958 (a D1 agonist). In contrast, the D2 antagonist RAC only attenuated amphetamine hyperactivity. Moreover, Baldo et al. (2002) found that ambulatory effects are blocked by injecting DA D1 and D2 antagonists into NAcSh, with a more prominent effect of DA D1 receptors than D2 on locomotor activity. Likewise, our data demonstrate that NPE induces locomotor activity via activation of both D1 and D2 receptors, but DA D1 receptors are necessary for the NPE-induced locomotion.

The food intake inhibition and increased locomotion induced by systemic administration of NPE were comparable to that induced by other amphetamine congeners, which are known to increase brain DA levels in the striatum (including the NAcSh), resulting in decreased food intake by promoting arousal, locomotion, and stereotypy (Kelley et al., 2005). Thus, it has been proposed that DA can be a neurotransmitter that mediates most pharmacological effects induced by appetite suppressants. Recently, it has been suggested that DA is also involved in the control of body weight, feeding, wakefulness, locomotion, and stereotypy (Seiden et al., 1993; Costa, 2007; Nicola, 2010; Tellez et al., 2012). Our results also suggest these appetite suppressants inhibited food intake, perhaps by promoting locomotion, a behavior that could compete with feeding (Kalyanasundar et al., 2015). To dissect the role of DA receptors, we blocked them, either systemically or intra NAcSh, and both yielded comparable results. Despite the limitations of restricting the diffusion of drugs at the NAcSh, our study points out DA receptors as important contributors to the NPE-induced locomotion and food intake suppression. Of course, our data did not preclude the participation of other brain regions in NPE’s effects. In this regard, the dorsal striatum would be an interesting target to explore its participation in the stereotypy induced by these appetite suppressants (Girasole et al., 2018; Engeln et al., 2020).

### NPE Evokes Neuronal Responses in the NAcSh

In the present study, we found that NPE modulated nearly 81.6% of NAcSh neurons recorded, either reducing (43.3%) or increasing (38.3%) their firing rates, with no differences in the percentage of inactive/active neurons (Figure 4C, *p* = n.s.). Despite the similar proportion of neurons modulated with either a positive or negative sign, the NAcSh population activity exhibited a net firing rate imbalance toward activation after NPE injection (that lasted < 30 min). Likewise, previous studies have shown that amphetamine, a DA releaser (Daberkow et al., 2013), activates some NAc neurons (Sombers et al., 2009). However, we also observed neurons with reduced responses after NPE. The inactive responses could be important to maintain the net inactivation/activation balance because, after 32 min, the population activity returned to BL activity levels; this is despite individual neurons continued responding over the 1 h of recordings. Accordingly, PCA trajectory analysis uncovered that NAcSh population activity, in fact, went into a dynamic pharmacological brain state (Figure 5). Given that the majority of cell types composing the NAcSh are GABAergic medium spiny neurons (either MSN-D1 or MSN-D2), it is most likely that NPE modulates them. We speculate that NPE activates MSN-D1 neurons because activation of these neurons is responsible for most, if not all, motor side effects induced by increasing DA levels in the striatum (Girasole et al., 2018; Engeln et al., 2020). However, and because our extracellular recordings could not distinguish among cell types, we do not know the identity of the cells that were either excited or inhibited or whether NPE affected NAcSh’s interneurons (Nicola and Malenka, 1997). Future studies should uncover what cell type(s) NPE is directly targeting.

### Comparison of NPE Versus Other Appetite Suppressants

Although NPE shares some similarities with other appetite suppressants, we also found major differences between them. A similarity was that all of them lead to weight loss, decreased food intake, and stimulated locomotor activity. Thus, NPE induces anorectic effects in the same manner as other phenethylamine derivatives such as diethylpropion, phentermine, bupropion, and cathinone; this is perhaps not surprising because chemically, these substances are all structurally related to amphetamine (Khan et al., 2012). Studies in humans and in rodents revealed that amphetamine congeners produce weight loss and decreased food intake at different levels with the following strength (amphetamine > cathinone > diethylpropion ≥ phentermine > NPE > bupropion) (Zelger and Carlini, 1980; Chen et al., 2001; Cercato et al., 2009; Hendricks et al., 2009; Kalyanasundar et al., 2015, 2016; Hauner et al., 2017; Lucchetta et al., 2017; Perez et al., 2019). In summary, what they all have in common is that their pharmacological effects on weight loss and food intake require DA signaling (Balcioglu and Wurtman, 1998; Baumann et al., 2000; Chen et al., 2001; Kalyanasundar et al., 2015; Lemieux et al., 2015). Another similarity among phentermine, diethylpropion, bupropion, and NPE is that they all promote an active awake state (reflecting insomnia) and also stimulate locomotion and produce stereotypy (Eisenberg et al., 1987; Kalyanasundar et al., 2015, 2016; Perez et al., 2019). They also modulate population NAcSh’s activity (Kalyanasundar et al., 2015; Perez et al., 2019). Although they modulate NAcSh activity, they do not seem to do it in the same manner (or magnitude). For example, a major difference is that diethylpropion, > phentermine, and > bupropion evoked a net inhibitory imbalance, respectively (Kalyanasundar et al., 2015). In contrast, here we found that NPE elicits a unique net NAcSh’s activation imbalance and a rapid return to population BL activity levels, not seen in the other appetite suppressants. The reason for these differences is not clear, but it can be hypothesized that it reflects the different degrees with which each appetite suppressant releases DA (Baumann et al., 2000; Rothman et al., 2001; Santamaría and Arias, 2010), as well as their effects in other neurotransmitters (e.g., norepinephrine, serotonin, and acetylcholine). Thus, the different neurochemical profiles of each appetite suppressant should determine its final modulatory pattern observed in the NAcSh population activity. Nevertheless, all these appetite suppressants share a common DA signaling in the NAcSh as an important component of most, if not all, amphetamine congeners to exert their anorectic and weight loss effects.

In summary, our results, in rats, provide evidence supporting a dopaminergic mechanism of action underlying the suppression of feeding and locomotion induced by NPE, which depends on its potency to release DA that in turn stimulates D1- and D2-like DA receptors in the NAcSh.

## Data Availability Statement

The raw data supporting the conclusions of this article will be made available by the authors, without undue reservation, to any qualified researcher.

## Ethics Statement

The animal study was reviewed and approved by the CINVESTAV institutional animal care and use committee.

## Author Contributions

BK and RG designed the research. MM and BK performed the research. CP, BA, and BK analyzed the data and made the figures. CP, BK, and RG wrote the article. All authors approved the final version of the manuscript.

## Conflict of Interest

The authors declare that the research was conducted in the absence of any commercial or financial relationships that could be construed as a potential conflict of interest.

## References

[B1] Alsiö J. Olszewski P. K. Norbäck A. H. Gunnarsson Z. E. A. Levine A. S. Pickering C. (2010). Dopamine D1 receptor gene expression decreases in the nucleus accumbens upon long-term exposure to palatable food and differs depending on diet-induced obesity phenotype in rats. *Neuroscience* 171 779–787. 10.1016/j.neuroscience.2010.09.046 20875839

[B2] Axel A. M. D. Mikkelsen J. D. Hansen H. H. (2010). Tesofensine, a novel triple monoamine reuptake inhibitor, induces appetite suppression by indirect stimulation of α1 adrenoceptor and dopamine D1 Receptor pathways in the diet-induced obese rat. *Neuropsychopharmacology* 35 1464–1476. 10.1038/npp.2010.16 20200509 PMC3055463

[B3] Balcioglu A. Wurtman R. J. (1998). Effects of phentermine on striatal dopamine and serotonin release in conscious rats: in vivo microdialysis study. *Int. J. Obes.* 22 325–328. 10.1038/sj.ijo.0800589 9578237

[B4] Baldo B. A. Sadeghian K. Basso A. M. Kelley A. E. (2002). Effects of selective dopamine D1 or D2 receptor blockade within nucleus accumbens subregions on ingestive behavior and associated motor activity. *Behav. Brain Res.* 137 165–177. 10.1016/S0166-4328(02)00293-0 12445722

[B5] Balint E. E. Falkay G. Balint G. A. (2009). Khat - a controversial plant. *Wien. Klin. Wochenschr.* 121 604–614. 10.1007/s00508-009-1259-7 19921126

[B6] Baumann M. H. Ayestas M. A. Dersch C. M. Brockington A. Rice K. C. Rothman R. B. (2000). Effects of phentermine and fenfluramine on extracellular dopamine and serotonin in rat nucleus accumbens: therapeutic implications. *Synapse* 36 102–113. 10.1002/(sici)1098-2396(200005)36:2<102::aid-syn3>3.0.co;2-# 10767057

[B7] Beninger R. J. (1983). The role of dopamine in locomotor activity and learning. *Brain Res. Rev.* 6 173–196. 10.1016/0165-0173(83)90038-3 6357357

[B8] Bray G. A. (2000). A concise review on the therapeutics of obesity. *Nutrition* 16 953–960. 10.1016/S0899-9007(00)00424-X 11054601

[B9] Brett E. M. (2019). “Pharmacotherapy for weight management,” in *Bariatric Endocrinology: Evaluation and Management of Adiposity, Adiposopathy and Related Diseases*, eds Gonzalez-Campoy J. M. Hurley D. L. Garvey W. T. (Cham: Springer International Publishing), 395–411. 10.1007/978-3-319-95655-8_21

[B10] Broening H. W. Morford L. L. Vorhees C. V. (2005). Interactions of dopamine D1 and D2 receptor antagonists with D-methamphetamine-induced hyperthermia and striatal dopamine and serotonin reductions. *Synapse* 56 84–93. 10.1002/syn.20130 15714503

[B11] Cauda F. Cavanna A. E. D’agata F. Sacco K. Duca S. Geminiani G. C. (2011). Functional connectivity and coactivation of the nucleus accumbens: a combined functional connectivity and structure-based meta-analysis. *J. Cogn. Neurosci.* 23 2864–2877. 10.1162/jocn.2011.21624 21265603

[B12] Cercato C. Roizenblatt V. A. Leança C. C. Segal A. Lopes Filho A. P. Mancini M. C. (2009). A randomized double-blind placebo-controlled study of the long-term efficacy and safety of diethylpropion in the treatment of obese subjects. *Int. J. Obes.* 33 857–865. 10.1038/ijo.2009.124 19564877

[B13] Chen T.-Y. Duh S.-L. Huang C.-C. Lin T.-B. Kuo D.-Y. (2001). Evidence for the involvement of dopamine D1 and D2 receptors in mediating the decrease of food intake during repeated treatment with amphetamine. *J. Biomed. Sci.* 8 462–466. 10.1007/BF02256608 11702009

[B14] Costa R. M. (2007). Plastic corticostriatal circuits for action learning. *Ann. N. Y. Acad. Sci.* 1104 172–191. 10.1196/annals.1390.015 17435119

[B15] Daberkow D. P. Brown H. D. Bunner K. D. Kraniotis S. A. Doellman M. A. Ragozzino M. E. (2013). Amphetamine paradoxically augments exocytotic dopamine release and phasic dopamine signals. *J. Neurosci.* 33 452–463. 10.1523/JNEUROSCI.2136-12.2013 23303926 PMC3711765

[B16] Derosa G. Maffioli P. (2012). Anti-obesity drugs: a review about their effects and their safety. *Expert Opin. Drug Saf.* 11 459–471. 10.1517/14740338.2012.675326 22439841

[B17] Dreher J. K. Jackson D. M. (1989). Role of D1 and D2 dopamine receptors in mediating locomotor activity elicited from the nucleus accumbens of rats. *Brain Res.* 487 267–277. 10.1016/0006-8993(89)90831-7 2525062

[B18] Drevets W. C. Gautier C. Price J. C. Kupfer D. J. Kinahan P. E. Grace A. A. (2001). Amphetamine-induced dopamine release in human ventral striatum correlates with euphoria. *Biol. Psychiatry* 49 81–96. 10.1016/S0006-3223(00)01038-6 11164755

[B19] Eddy N. B. Halbach H. Isbell H. Seevers M. H. (1965). Drug dependence: its significance and characteristics. *Bull. World Health Organ.* 32 721–733. 5294186 PMC2555251

[B20] Eisenberg M. S. Maher T. J. Silverman H. I. (1987). A comparison of the effects of phenylpropanolamine, d-amphetamine and d-norpseudoephedrine on open-field locomotion and food intake in the rat. *Appetite* 9 31–37. 10.1016/0195-6663(87)90051-1 3662492

[B21] Engeln M. Song Y. Chandra R. La A. Fox M. E. Evans B. (2020). Individual differences in stereotypy and neuron subtype translatome with TrkB deletion. *bioRxiv* [Preprint], 10.1038/s41380-020-0746-0 32366954 PMC8480032

[B22] Fernandes A. B. Alves da Silva J. Almeida J. Cui G. Gerfen C. R. Costa R. M. (2020). Postingestive modulation of food seeking depends on vagus-mediated dopamine neuron activity. *Neuron* 106 778–788.e6. 10.1016/j.neuron.2020.03.009 32259476 PMC7710496

[B23] Gadagkar S. R. Call G. B. (2015). Computational tools for fitting the Hill equation to dose-response curves. *J. Pharmacol. Toxicol. Methods* 71 68–76. 10.1016/j.vascn.2014.08.006 25157754

[B24] Geiger B. M. Haburcak M. Avena N. M. Moyer M. C. Hoebel B. G. Pothos E. N. (2009). Deficits of mesolimbic dopamine neurotransmission in rat dietary obesity. *Neuroscience* 159 1193–1199. 10.1016/j.neuroscience.2009.02.007 19409204 PMC2677693

[B25] Gervasoni D. Lin S.-C. Ribeiro S. Soares E. S. Pantoja J. Nicolelis M. A. L. (2004). Global forebrain dynamics predict rat behavioral states and their transitions. *J. Neurosci.* 24 11137–11147. 10.1523/JNEUROSCI.3524-04.2004 15590930 PMC6730270

[B26] Girasole A. E. Lum M. Y. Nathaniel D. Bair-Marshall C. J. Guenthner C. J. Luo L. (2018). A Subpopulation of striatal neurons mediates levodopa-induced Dyskinesia. *Neuron* 97 787–795.e6. 10.1016/j.neuron.2018.01.017 29398356 PMC6233726

[B27] Greenway F. L. (1992). Clinical studies with phenylpropanolamine: a metaanalysis. *Am. J. Clin. Nutr.* 55 203S–205S. 10.1093/ajcn/55.1.203s 1530830

[B28] Gutiérrez R. Rodriguez-Ortiz C. J. De La Cruz V. Núñez-Jaramillo L. Bermudez-Rattoni F. (2003). Cholinergic dependence of taste memory formation: evidence of two distinct processes. *Neurobiol. Learn. Mem.* 80 323–331. 10.1016/S1074-7427(03)00066-2 14521874

[B29] Gutierrez R. Simon S. A. Nicolelis M. A. L. (2010). Licking-induced synchrony in the taste-reward circuit improves cue discrimination during learning. *J. Neurosci.* 30 287–303. 10.1523/JNEUROSCI.0855-09.2010 20053910 PMC2831544

[B30] Hansen H. H. Jensen M. M. Overgaard A. Weikop P. Mikkelsen J. D. (2013). Tesofensine induces appetite suppression and weight loss with reversal of low forebrain dopamine levels in the diet-induced obese rat. *Pharmacol. Biochem. Behav.* 110 265–271. 10.1016/j.pbb.2013.07.018 23932919

[B31] Harris S. C. Ivy A. C. Searle L. M. (1947). The mechanism of amphetamine-induced loss of weight: a consideration of the theory of hunger and appetite. *J. Am. Med. Assoc.* 134 1468–1475. 10.1001/jama.1947.02880340022005 20255617

[B32] Hauner H. Hastreiter L. Werdier D. Chen-Stute A. Scholze J. Blüher M. (2017). Efficacy and safety of cathine (Nor-Pseudoephedrine) in the treatment of obesity: a randomized dose-finding study. *Obes. Facts* 10 407–419. 10.1159/000478098 28873376 PMC5644935

[B33] Hendricks E. J. Rothman R. B. Greenway F. L. (2009). How physician obesity specialists use drugs to treat obesity. *Obesity* 17 1730–1735. 10.1038/oby.2009.69 19300434

[B34] Hoffman D. C. Beninger R. J. (1985). The D1 dopamine receptor antagonist, SCH 23390 reduces locomotor activity and rearing in rats. *Pharmacol. Biochem. Behav.* 22 341–342. 10.1016/0091-3057(85)90401-0 2858871

[B35] Joo J. K. Lee K. S. (2014). Pharmacotherapy for obesity. *J. Menopausal Med.* 20 90–96. 10.6118/jmm.2014.20.3.90 25580419 PMC4286660

[B36] Kalix P. (1992). Cathinone, a natural amphetamine. *Pharmacol. Toxicol.* 70 77–86. 10.1111/j.1600-0773.1992.tb00434.x 1508843

[B37] Kalix P. (1996). *Catha edulis*, a plant that has amphetamine effects. *Pharm. World Sci.* 18 69–73. 10.1007/BF00579708 8739260

[B38] Kalix P. Geisshusler S. Brenneisen R. Koelbing U. Fisch H. U. (1990). Cathinone, a phenylpropylamine alkaolid from khat leaves that has amphetamine effects in humans. *NIDA Res. Monogr.* 105 289–290. 1876014

[B39] Kalix P. Khan I. (1984). Khat: an amphetamine-like plant material. *Bull. World Health Organ.* 62 681–686. 6334569 PMC2536214

[B40] Kalyanasundar B. Perez C. I. Luna A. Solorio J. Moreno M. G. Elias D. (2015). D1 and D2 antagonists reverse the effects of appetite suppressants on weight loss, food intake, locomotion, and rebalance spiking inhibition in the rat NAc shell. *J. Neurophysiol.* 114 585–607. 10.1152/jn.00012.2015 25972577 PMC4509405

[B41] Kalyanasundar B. Solorio J. Perez C. I. Hoyo-Vadillo C. Simon S. A. Gutierrez R. (2016). The efficacy of the appetite suppressant, diethylpropion, is dependent on both when it is given (day vs. night) and under conditions of high fat dietary restriction. *Appetite* 100 152–161. 10.1016/j.appet.2016.01.036 26867698

[B42] Kelley A. E. Baldo B. A. Pratt W. E. Will M. J. (2005). Corticostriatal-hypothalamic circuitry and food motivation: integration of energy, action and reward. *Physiol. Behav.* 86 773–795. 10.1016/j.physbeh.2005.08.066 16289609

[B43] Khan J. I. Kennedy T. J. Christian D. R. (2012). “Phenethylamines,” in *Basic Principles of Forensic Chemistry*, eds Khan J. I. Kennedy T. J. Christian D. R. Jr. (Totowa, NJ: Humana Press), 157–176. 10.1007/978-1-59745-437-7_13

[B44] Knab A. M. Bowen R. S. Hamilton A. T. Lightfoot J. T. (2012). Pharmacological manipulation of the dopaminergic system affects wheel-running activity in differentially active mice. *J. Biol. Regul. Homeost. Agents* 26 119–129. 22475103 PMC4190615

[B45] Kushner R. F. (2018). Weight loss strategies for treatment of obesity: lifestyle management and pharmacotherapy. *Prog. Cardiovasc. Dis.* 61 246–252. 10.1016/j.pcad.2018.06.001 29890171

[B46] Lecca D. Piras G. Driscoll P. Giorgi O. Corda M. G. (2004). A differential activation of dopamine output in the shell and core of the nucleus accumbens is associated with the motor responses to addictive drugs: a brain dialysis study in Roman high- and low-avoidance rats. *Neuropharmacology* 46 688–699. 10.1016/j.neuropharm.2003.11.011 14996546

[B47] Lemieux A. M. Li B. Al’Absi M. (2015). Khat use and appetite: an overview and comparison of amphetamine, khat and cathinone. *J. Ethnopharmacol.* 160 78–85. 10.1016/j.jep.2014.11.002 25435289 PMC4281284

[B48] Liu X. Strecker R. E. Brener J. (1998). Dopamine depletion in nucleus accumbens influences locomotion but not force and timing of operant responding. *Pharmacol. Biochem. Behav.* 59 737–745. 10.1016/S0091-3057(97)00547-549 9512080

[B49] Lucchetta R. C. Riveros B. S. Pontarolo R. Radominski R. B. Otuki M. F. Fernandez-Llimos F. (2017). Systematic review and meta-analysis of the efficacy and safety of amfepramone and mazindol as a monotherapy for the treatment of obese or overweight patients. *Clinics* 72 317–324. 10.6061/clinics/2017(05)10 28591345 PMC5439101

[B50] Meyer M. E. Cottrell G. A. van Hartesveldt C. Potter T. J. (1993). Effects of dopamine D1 antagonists SCH23390 and SK&F83566 on locomotor activities in rats. *Pharmacol. Biochem. Behav.* 44 429–432. 10.1016/0091-3057(93)90486-D 8446676

[B51] Moratalla R. Khairnar A. Simola N. Granado N. García-Montes J. R. Porceddu P. F. (2017). Amphetamine-related drugs neurotoxicity in humans and in experimental animals: main mechanisms. *Prog. Neurobiol.* 155 149–170. 10.1016/j.pneurobio.2015.09.011 26455459

[B52] Nencini P. Fraioli S. Perrella D. (1996). Tolerance does not develop to the suppressant effects of (−)-norpseudoephedrine on ingestive behavior in the rat. *Pharmacol. Biochem. Behav.* 53 297–301. 10.1016/0091-3057(95)02024-2021 8808135

[B53] Nicola S. M. (2010). The flexible approach hypothesis: unification of effort and cue-responding hypotheses for the role of nucleus Accumbens dopamine in the activation of reward-seeking behavior. *J. Neurosci.* 30 16585–16600. 10.1523/JNEUROSCI.3958-10.2010 21147998 PMC3030450

[B54] Nicola S. M. Malenka R. C. (1997). Dopamine depresses excitatory and inhibitory synaptic transmission by distinct mechanisms in the nucleus Accumbens. *J. Neurosci.* 17 5697–5710. 10.1523/JNEUROSCI.17-15-05697.1997 9221769 PMC6573215

[B55] Onakpoya I. J. Heneghan C. J. Aronson J. K. (2016). Post-marketing withdrawal of anti-obesity medicinal products because of adverse drug reactions: a systematic review. *BMC Med.* 14:191. 10.1186/s12916-016-0735-y 27894343 PMC5126837

[B56] O’Neill M. F. Shaw G. (1999). Comparison of dopamine receptor antagonists on hyperlocomotion induced by cocaine, amphetamine, MK-801 and the dopamine D1 agonist C-APB in mice. *Psychopharmacology* 145 237–250. 10.1007/s002130051055 10494572

[B57] Palmiter R. D. (2007). Is dopamine a physiologically relevant mediator of feeding behavior? *Trends Neurosci.* 30 375–381. 10.1016/j.tins.2007.06.004 17604133

[B58] Pehek E. A. Schechter M. D. Yamamoto B. K. (1990). Effects of cathinone and amphetamine on the neurochemistry of dopamine in vivo. *Neuropharmacology* 29 1171–1176. 10.1016/0028-3908(90)90041-O 2293059

[B59] Perez C. I. Kalyanasundar B. Moreno M. G. Gutierrez R. (2019). The triple combination Phentermine Plus 5-HTP/carbidopa leads to greater weight loss, with fewer psychomotor side effects than each drug alone. *Front. Pharmacol.* 10:1372. 10.3389/fphar.2019.01327 31780943 PMC6851240

[B60] Peterson D. W. Maitai C. K. Sparber S. B. (1980). Relative potencies of two phenylalkylamines found in the abused plant *Catha edulis*, khat. *Life Sci.* 27 2143–2147. 10.1016/0024-3205(80)90496-8 6111010

[B61] Powell E. W. Leman R. B. (1976). Connections of the nucleus accumbens. *Brain Res.* 105 389–403. 10.1016/0006-8993(76)90589-8 816427

[B62] Reimer A. R. Martin-Iverson M. T. Urichuk L. J. Coutts R. T. Byrne A. (1995). Conditioned place preferences, conditioned locomotion, and behavioral sensitization occur in rats treated with diethylpropion. *Pharmacol. Biochem. Behav.* 51 89–96. 10.1016/0091-3057(94)00364-O 7617738

[B63] Richert L. (2011). Trimming down: the debate over weight loss drugs and the push for a leaner FDA, 1979-2001. *Pharm. Hist.* 53 55–69. 23045876

[B64] Rothman R. B. Baumann M. H. (2006). Balance between dopamine and serotonin release modulates behavioral effects of amphetamine-type drugs. *Ann. N. Y. Acad. Sci.* 1074 245–260. 10.1196/annals.1369.064 17105921

[B65] Rothman R. B. Baumann M. H. Dersch C. M. Romero D. V. Rice K. C. Carroll F. I. (2001). Amphetamine-type central nervous system stimulants release norepinephrine more potently than they release dopamine and serotonin. *Synapse* 39 32–41. 10.1002/1098-2396(20010101)39:1<32::aid-syn5>3.0.co;2-3 11071707

[B66] Santamaría A. Arias H. R. (2010). Neurochemical and behavioral effects elicited by bupropion and diethylpropion in rats. *Behav. Brain Res.* 211 132–139. 10.1016/j.bbr.2010.03.023 20307582

[B67] Schechter M. D. (1990a). Dopaminergic nature of acute cathine tolerance. *Pharmacol. Biochem. Behav.* 36 817–820. 10.1016/0091-3057(90)90083-T 1977178

[B68] Schechter M. D. (1990b). Rats become acutely tolerant to cathine after amphetamine or cathinone administration. *Psychopharmacology* 101 126–131. 10.1007/BF02253729 1971444

[B69] Schwienbacher I. Fendt M. Hauber W. Koch M. (2002). Dopamine D1 receptors and adenosine A1 receptors in the rat nucleus accumbens regulate motor activity but not prepulse inhibition. *Eur. J. Pharmacol.* 444 161–169. 10.1016/S0014-2999(02)01622-9 12063076

[B70] Segal D. S. Mandell A. J. (1974). Long-term administration of d-amphetamine: progressive augmentation of motor activity and stereotypy. *Pharmacol. Biochem. Behav.* 2 249–255. 10.1016/0091-3057(74)90060-4 4857295

[B71] Seiden L. S. Sabol K. E. Ricaurte G. A. (1993). Amphetamine: effects on catecholamine systems and behavior. *Annu. Rev. Pharmacol. Toxicol.* 33 639–676. 10.1146/annurev.pa.33.040193.003231 8494354

[B72] Sharp J. C. Nielson H. C. Porter P. B. (1962). The effect of amphetamine upon cats with lesions in the ventromedial hypothalamus. *J. Comp. Physiol. Psychol.* 55 198–200. 10.1037/h0041160 13911409

[B73] Sombers L. A. Beyene M. Carelli R. M. Wightman R. M. (2009). Synaptic overflow of dopamine in the nucleus accumbens arises from neuronal activity in the ventral Tegmental area. *J. Neurosci.* 29 1735–1742. 10.1523/JNEUROSCI.5562-08.2009 19211880 PMC2673986

[B74] Stark P. Totty C. W. (1967). Effects of amphetamines on eating elicited by hypothalamic stimulation. *J. Pharmacol. Exp. Ther.* 158 272–278. 6065150

[B75] Stowe F. R. Miller A. T. (1957). The effect of amphetamine on food intake in rats with hypothalamic hyperphagia. *Experientia* 13 114–115. 10.1007/BF02157566 13427727

[B76] Tellez L. A. Perez I. O. Simon S. A. Gutierrez R. (2012). Transitions between sleep and feeding states in rat ventral striatum neurons. *J. Neurophysiol.* 108 1739–1751. 10.1152/jn.00394.2012 22745464 PMC3544947

[B77] Thiel A. Dressler D. (1994). Dyskinesias possibly induced by norpseudoephedrine. *J. Neurol.* 241 167–169. 10.1007/BF00868344 8164019

[B78] Toennes S. W. Harder S. Schramm M. Niess C. Kauert G. F. (2003). Pharmacokinetics of cathinone, cathine and norephedrine after the chewing of khat leaves. *Br. J. Clin. Pharmacol.* 56 125–130. 10.1046/j.1365-2125.2003.01834.x 12848785 PMC1884326

[B79] Wing R. R. Hill J. O. (2001). Successful weight loss maintenance. *Annu. Rev. Nutr.* 21 323–341. 10.1146/annurev.nutr.21.1.323 11375440

[B80] Wright J. M. Dobosiewicz M. R. S. Clarke P. B. S. (2013). The role of dopaminergic transmission through D1-like and D2-like receptors in amphetamine-induced rat ultrasonic vocalizations. *Psychopharmacology* 225 853–868. 10.1007/s00213-012-2871-1 23052567

[B81] Zelger J. L. Carlini E. A. (1980). Anorexigenic effects of two amines obtained from *Catha edulis* Forsk (Khat) in rats. *Pharmacol. Biochem. Behav.* 12 701–705. 10.1016/0091-3057(80)90152-5 7393964

