# Supplementary material for: The Appetite Suppressant D-norpseudoephedrine (Cathine) Acts via D1/D2-Like Dopamine Receptors in the Nucleus Accumbens Shell
Source: Front Neurosci. 2020 Oct 16;14:572328. doi: 10.3389/fnins.2020.572328 (PMC7596745; doi:10.3389/fnins.2020.572328)
Supplement: Supplementary file 2 [file Table_2.DOCX]

Supplementary Material

# Supplementary Figures


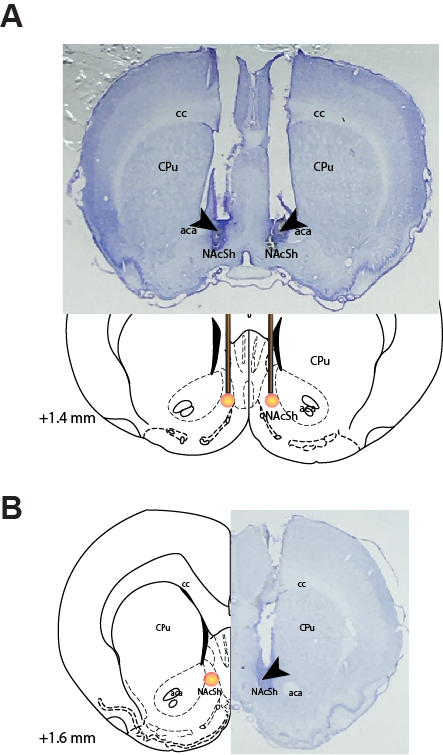


**Supplementary Figure 1.** Histological identification depicting representative canulae and recording sites in NAcSh. **(A)** Nissl-stained coronal section showing bilateral cannulae in NAcSh indicated by the black arrowhead, aligned to the nearest section adaptation from Paxinos & Watson rat brain atlas (1998). **(B)** Nissl-stained coronal section showing an example electrode track indicated by the black arrow. NAcSh, nucleus accumbens shell; CPu, caudate putamen (striatum); cc, corpus callosum; aca, anterior commissure.
